# Medico-Chirurgical Transactions, Vol. XIII. Concluded

**Published:** 1827-04

**Authors:** 


### MEDICO-CHIRURGICAL TRANSACTIONS.

[Vol. XIII. Concluded.]

### Art. I.

### *Hydrometra and Dry Gangrene.* Dr. Thomson.

Before we make any remarks on Dr. Thomson's case, we shall lay the particulars of it before our readers.

Mary Rae, aged 65, was admitted a patient in the Infirmary of the Chelsea Work-house, April 1, 1823, having previously enjoyed good health, and reported herself of temperate habits till very lately, when grief for the loss of a husband induced her to take to the use of spirituous liquors. She had borne two children. On admission, she laboured under acute rheumatism, for which she was bled, purged, and took colchicum and oil of turpentine. In two months she was discharged convalescent; but, in five months afterwards she returned, being emaciated, and complaining of pain connected with a tumour in the abdomen, which she had perceived six weeks previously. This tumour was situated in the lower part of the abdominal cavity, rising as it were out of the pelvis, and occupying the iliac, hypogastric, and umbilical regions. She appeared as if six months gone with child. An indistinct fluctuation was perceptible, and the least external pressure excited pain. It was suspected that the case was a diseased ovarium; but the accompanying symptoms denoted a greater derangement of the system than usually attends ovarian dropsy. These were, want of appetite, nausea, furred tongue, quick pulse, irregular bowels, scanty and high-coloured urine. She was confined to bed, and put on vegetable diet. Mercurials, so as to affect the mouth, were also prescribed, and the bowels kept open. Leeches, blisters, &c. were employed. The increase of the swelling was prevented, but the sensation of pain, on pressure, remained. In this state she continued till the middle of January, 1824, when the left foot was found to be affected with dry gangrene, which gradually extended to the knee. As a feeble pulsation could be felt in the popliteal artery, it was deemed advisable to amputate, a line of separation having been formed between the sound and gangrened parts. But the operation was delayed in consequence of the reluctance of the patient, till the 29th February, when it was performed four inches above the knee. Every thing appeared to be going on well till the third day, when she suddenly sunk exhausted.

*Dissection*. On laying open the abdomen, a body resembling the gravid uterus, occupying the whole pelvic and much of the abdominal cavity, was discovered, on the anterior surface of which was the urinary bladder, so that it must have been perforated, if a trochar had been employed to evacuate the fluid during life. The tumour was easily ascertained to be the uterus filled with fluid. It was partially sphacelated in its peritoneal covering. The stomach, colon, and other intestines, were glued together in many places, and in some were sphacelated. The uterus was found to contain eight quarts of liquid, of a dark brown colour, and slightly coagulable by heat. The os uteri was completely obliterated—the ovaria small and flaccid, but otherwise natural. The aorta was found to be ossified about two inches above its division into the iliacs. The right iliacs were healthy, but the left were ossified in several places.

Dr. Thomson makes some physiological and pathological remarks on this case, which we may shortly notice. He attributes the gangrene to the ossification in the large trunks, impeding the circulation. We confess we cannot see how a ring of ossification in the aorta or in the iliacs, should prevent a due supply of blood in the parts below the said ossifications.

In respect to the hydrometra, it is rather a rare disease in its pure and unmixed state. Dr. Denman, indeed, has doubted the existence of such a disease, concluding that what is called hydrometra, is probably no more than one large hydatid. The case in question, however, is a proof that such a disease can exist.

### ART. II.

*Cases of Destructive Inflammation of the Eye, and of Suppurative Inflammation of the Integuments, occurring in the Puerperal State, and apparently from Constitutional Causes.* By MARSHALL HALL, M.D. and JOHN HIGGINBOTTOM, Esq.

It occurred to these two gentlemen to witness together, at distant periods, the progress of several cases of a singular morbid affection about to be described. It took place at from five to eleven days after delivery, and was always preceded by some serious indisposition. In one case there were all the symptoms of intestinal irritation and of exhaustion from uterine hæmorrhage—in a second, there was protracted diarrhœa—and in the others much fever, with functional derangement of the bowels. In one remarkable circumstance, however, they all concurred—the left eye, and not the right, was always the one affected.

"In one case the eye was affected but a day or two, and but slightly, when the patient died. In two there was great chemosis, the transparency of the cornea was destroyed, and the eye appeared collapsed, during life; and in a fourth the patient survived the ulceration and sloughing of the cornea, the total destruction of the organ, and the subsequent healing of its anterior part.

"Soon after the appearance of the disease in the eye, there has also appeared a local inflammation situated in the integuments, and first observed on the hand, but found, on a careful examination, either at the same time, or soon afterwards, on the inferior as well as superior extremities. In one of the cases only, there was no such cutaneous inflammation; but the patient having been bled, there were inflammation and suppuration of the vein. In two of the cases the cutaneous inflammation was observed on the hands and feet, rather diffuse and little elevated, but extremely tender to the touch; in the fourth, that in which the patient survived the loss of the eye, the patches of inflammation were very numerous and extensive, and led to equally numerous and extensive formations of pus.

"All remedies appeared to be unavailing. Early bleeding, mercury, and purgatives, and afterwards bark and opium, were fully tried without effect. The sinking of the vital powers alone appeared to suspend the course of this terrible disease.

"From the preceding observations it may be conjectured that this morbid affection has a constitutional origin. This inference seems warranted by all the patients having previously experienced some indisposition, chiefly a deranged state of the function of the bowels, and by their all suffering from more than one local disease at the same time." 191.

Five cases are given in illustration. We can only spare room for the particulars of one, as an example.

*Case.* Mrs. B. aged 27, of sanguineous temperament, was safely delivered, on the 13th November, 1823, without much hæmorrhagy. On the second day she became affected with fever and derangement of the bowels, which continued till the eleventh day, on the morning of which, a slight redness of the left eye was discernible, confined to the external canthus. At the same time a slight blush of redness was observed on the back of the right hand, and afterward along the ulna. In the evening the eye was affected with slight chemosis, the conjunctiva protruding in a loose flabby state, and with scarce any redness. On the succeeding morning, all the morbid appearances were augmented—the chemosis was extensive, the cornea was clear, but covered with a slight film of mucus—no appearance of ulceration at the junction of the cornea with the tunica conjunctiva—the eyelid was slightly swollen—the pupil not contracted. The inflamed parts of the hand and arm were extremely red and painful, with slight fluctuation. The patient soon sunk.

In a postcript, Dr. Hall informs us that Mr. Ward, surgeon of Olerton, observed a similar instance in his own practice. It also occurred after a tedious labour and much hæmorrhage. In this case the eye-ball burst, and the patient soon died.

We are totally at a loss to account for such an occurrence as that described by the gentlemen above-mentioned. The circumstance, too, of the left eye being invariably affected, is perfectly inscrutable.

### Art. III.

*Some Particulars of a remarkable Disease of the Heart, attended with Partial Discoloration of the Skin.* By James Johnson, M.D.

As this case is short, and very curious, we shall give it in the words of the author, especially as no comments can be made upon it in this Journal.

"Mrs. A. aged about 30 years, had enjoyed good health till the birth of her first and only child, about seven years ago. From that time she had often been ailing, though she could give no distinct or satisfactory account of her symptoms, they being such as are generally denominated nervous or hysterical. About eighteen months ago, however, her health got seriously worse, after an attack of inflammation about the womb and lower part of the abdomen. About this time a singular change took place in the colour of the skin, in all those parts (and those only) which are usually exposed to the air and light, viz. her face, neck, and hands. The colour of those parts resembled that of a very dark Mulatto, though the skin of all other parts of the body was remarkably fair and delicate.

"When I first saw Mrs. A. about a month before her death, she presented the following symptoms. Her appetite was voracious, being hungry in the course of a few hours after a hearty meal. She had a sensation of sinking at the stomach when empty, but no distinct pain. She was easily put out of breath by any corporeal exertion, though she could expand her chest, and take in a very large volume of air without the least difficulty. Her bowels were not disordered, and the secretions were natural. Her muscular strength was exceedingly impaired, notwithstanding her voracious appetite, and she felt an indescribable malaise and restlessness, for which she could assign no reason. Her tongue had a very remarkable appearance, being rough and chapped. Her pulse was habitually so feeble, as to be with difficulty distinguished at the wrist; but her most distressing complaint was what she called 'fits of heaving,' quite different from sickness of stomach, and never attended with vomiting or any discharge from that viscus. I saw her in one of these fits, and it appeared more allied to globus hystericus than any thing that I could compare it with. These fits were attended with faintness, and once or twice with syncope. She had consulted many medical men, and taken various medicines, but with little or no benefit.

"I examined the thorax and abdomen very minutely, but could discover no proof of disease in either of these quarters. The chest sounded well in every part, and the respiratory murmur could be heard every where by the naked ear and by the stethoscope. The pulsations of the heart, however, could not be felt or heard in any part of the chest.

"With the warm weather, in the early part of June, her debility made rapid progress, and the 'fits of heaving' became more frequent and distressing. She now lost her appetite, and on the 19th of June she expired, after suffering dreadful restlessness, constant jactitation, and sense of unutterable distress internally, for three or four days previous to her decease.

"Being often pressed for an opinion respecting the nature of the malady by her husband (an eminent barrister), and by her friends, I stated my conviction that there was some organic disease of the heart, the precise nature of which I could not determine. I was requested by Serjeant A. to examine the body, which I did in the presence of Mr. Haines and Mr. Evans, surgeons at Hampstead, on the 20th of June. To them I reiterated the above opinion before the dissection.

"*Examination.*—Every organ and structure in the body appeared in a state of the most perfect integrity, except the heart. The *right* ventricle of the heart was enlarged in its capacity, but its parietes were exceedingly extenuated, being not thicker, in any part, than the seventh or eighth of an inch. The parietes of the *left* ventricle, on the contrary, were full three quarters of an inch in thickness, and the capacity of this chamber was so much lessened that it would scarcely admit the forefinger. There was nothing remarkable in the valves, or in the great vessels leading from the heart, with the exception of the cava inferior, which was of a most extraordinary size.

"*Remarks.*—It must be obvious that all equilibrium between the two sides of the heart was destroyed, and that the important function of the circulation was carried on in a most imperfect manner. It appeared to the medical gentlemen present, and to myself, that the left ventricle was incapable of throwing out more than two or three drachms of blood at each systole; and this explains the extreme smallness and feebleness of the pulse, as well as the general debility. The difficulty which such a heart must have experienced in transmitting the blood from the lungs to the general circulation, may also explain the breathlessness on any muscular exertion; but the change of colour in the skin, and the distressing *heavings* are phenomena which are not so easily accounted for.

"As this was a case which excited great interest, and produced much contrariety of opinion and speculation among medical men, during the life of the amiable patient, I trust the *post mortem* examination may not be entirely devoid of interest, or even of practical utility.

"My principal object in submitting these notes of the case to the consideration of the Society, is to draw their attention to the very numerous and anomalous symptoms produced by a defect in the central organ of the circulation; symptoms which are very frequently referred to other sources, when not elucidated by rigorous examination after death." 216.

### Art. IV.

### *On the Medical Properties of the Subcarbonate of Iron.* By John Elliotson, M.D. Cantab. &c. &c.

Our readers are aware that Dr. Elliotson made some experiments and published them, on large doses of the pulvis antimonialis. In this paper, he proposes to shew that very large quantities of the carbonate of iron may be taken also, without producing any inconvenience, but with decidedly good effects. The usual dose of this medicine is from fifteen to thirty grains twice or thrice a day. Many, however, have lately exhibited it in drachm doses, or even larger, since the medicine has become so useful in neuralgic affections. Dr. E. has ascertained, by numerous trials, that it may be given in doses varying from two to four drachms every six—nay, every four hours—that the dose of half an ounce may be commenced with at first, without any preparation, and may be continued many weeks, without inconvenience. However large the dose, neither headache, thirst, heat, foulness of tongue, griping, nor constipation followed, or were increased if previously present. But the question occurs, is there any advantage from these large doses over smaller ones? This question our author is unable to answer. "A drachm dose may perhaps be as useful as one of half an ounce."

"Reasoning analogically, one would conceive that large quantities must be more serviceable. Cinchona, for instance, will cure an ague in doses of a drachm, given every two hours, when two or three table-spoonfuls of the decoction three times a-day, or even a drachm of the substance given not more frequently, altogether fails. We are accustomed to increase the doses of all medicines for the purpose of increasing the effect. In the very example of subcarbonate of iron, practitioners prescribe sometimes what they consider a small dose, sometimes what they consider a large one, according to the force which they judge necessary to bring up against the disease; and they increase the quantity as the disease proves obstinate. Now since it appears that the dose of this medicine has a much wider range than has been thonght, and that the terms large and small must relate to quantities different from those to which they have been hitherto applied, the same habit of practice would incline one to these large doses in obstinate cases, unless such comparative observations, as have been just alluded to, prove them to be no wise superior to what have hitherto been considered large." 238.

Cullen seems to have been of opinion that the good effects of preparations of iron have often been missed by the medicine being given in too small doses. And Sydenham was in the habit of giving the simple rust of iron freely. It is curious that Cullen mentions the rust to have been given in the quantity of six drachms in one day; but observes that he had found hardly any stomach capable of bearing more than a third of that dose. Before concluding these preliminary observations, Dr. E. begs to say that a scruple of the sulphate of iron, made into pills, with the extract of gentian, is borne just as well as half an ounce of the subcarbonate—and that every observation which he has made respecting this dose of the *latter* applies nearly to that of the *former*.

Eight cases are related by Dr. Elliotson in illustration, of which we shall give the particulars of two.

*Case* 1. This was one of paralysis agitans, in which Dr. E. tried the subcarbonate, after first having brought out a crop of pustules on the limbs by the tartar-emetic ointment, and purged him freely with oil of turpentine. He also went through a course of the sulphate of zinc, and was blistered on the occiput. Dr. E. then prescribed the iron in half drachm doses every eight hours, with leeches to the temples, and a perpetual blister to the forehead. The dose was gradually increased until the patient took three drachms for a dose, when the shaking was greatly abated, and the man finally left the hospital perfectly well.

*Case* 2. The first case induced our author to try the same medicine in chorea, in the person of an emaciated pale girl of 14 years, who had universal chorea. It was one of the most frightful cases our author ever saw, she being unable to walk or stand, and could scarcely be kept in bed without the assistance of two or three persons. She was continually screaming —never slept—and had suffered some epileptic fits. She was first purged with the submuriate of mercury, and then the subcarbonate of iron was commenced in half-drachm doses every six hours. It was gradually increased to a drachm, and in six weeks she was discharged cured.

All the remaining cases are those of chorea, and in all our author was successful. Yet Dr. E. does not attempt to extol the medicine as a specific. He is confident the iron may be often given safely when there are head-aches, foul-tongue, and torpid bowels; it being prudent, however, in such cases, to draw blood, and open the bowels. Dr. E. has failed with the largest quantities of iron in epilepsy, cancer, and lupus; but found it very beneficial in chronic neuralgia, and various chronic ulcerations and pustular diseases, as well as those diseases of debility in which it is so justly celebrated. Dr. E. gives a case where the sulphate was used in chorea, instead of the carbonate, and with beneficial effects. Dr. Elliotson deserves the thanks of the profession for his zeal and ability in trying the remedial efficacy of certain medicaments in unusual doses. As far, however, as regards the carbonate of iron, we are disposed to think that, when very large doses are given, a great proportion of the medicine passes the stomach and bowels unchanged, and without doing more than much smaller quantities.

### Art. V.

### *Notes of a Case of Hydrophobia, with some Remarks on the Pathology of that Disease.* By Geo. Gregory, M.D.

The phenomena of this case, during the miserable life of the patient, present nothing very particular. On dissection, the spinal marrow was found free from disease throughout its whole extent. There was some slight effusion on the surface and in the ventricles of the brain. The stomach and bowels were in a healthy state—lungs gorged with blood. The internal surface of the pharynx, epiglottis, larynx, œsophagus, and trachea, appeared of a coffee-ground colour, or almost black, which colour remained after the parts were sponged. There was no breach of structure in any of these parts. The inner coat of the aorta, and large arteries proceeding from it, was of a bright scarlet colour. Heart and pericardium healthy.

Dr. Gregory observes that, although the appearances on dissection, in hydrophobia, vary exceedingly, yet in this case, at least, it is impossible not to connect the symptoms with the appearances about the throat, on dissection. "These phenomena tend to the conclusion, that the symptom which gives name to the disease is directly dependent upon some form of inflammatory action in the larynx and pharynx, and that the true nosological situation of hydrophobia is in the genus cynanche." The practical inference which our author would draw from these data is, "that when a man has been bitten by a rabid animal, he should be closely watched about the end of the fourth week, and on the first appearance of any marks of nervous irritation or uneasiness about the throat, those remedies should be adopted, of whatever kind, which the practitioner would have employed, had the real nature of the disease been unequivocally ascertained." We fear that it is upon the *prophylactic* treatment we are solely to rely in the present state of our knowledge of hydrophobia. That prophylaxis, we imagine, will hinge on cupping the wound well—then excising it, and establishing a suppuration by means of caustic.

It is proper to state, however, that, in a paper by Mr. Hewitt, of the Bombay Medical Establishment, on the bites of a mad jackall, one patient was apparently saved, after the hydrophobic symptoms had commenced, by rapidly inducing ptyalism. This corresponds with the experience of Mr. Daniel Johnson, as published some years ago in this Journal.

We have now finished the present volume of the Medico-Chirurgical Transactions, and presented our readers with almost every fact contained in it.

We shall look with impatience for another production from the same source, and hope it will not prove inferior to its predecessors.

